# Research on Combined Localization Algorithm Based on Active Screening–Kalman Filtering

**DOI:** 10.3390/s24072372

**Published:** 2024-04-08

**Authors:** Xiao Zhang, Yuting Fu, Jie Li, Yandong Wei, Yu Li, Lu Zheng

**Affiliations:** 1 College of Engineering, China Agricultural University, Beijing 100083, China; x.zhang@cau.edu.cn (X.Z.); yutingfu@cau.edu.cn (Y.F.); lijie090@cau.edu.cn (J.L.); liyu22@cau.edu.cn (Y.L.); 2 Industrial Technology Centre, Hebei Petroleum University of Technology, Chengde 067000, China; cdpc_wyd@cdpc.edu.cn

**Keywords:** active filtering, combined positioning, Kalman filtering, static positioning, dynamic positioning

## Abstract

Real-time acquisition of location information for agricultural robotic systems is a prerequisite for achieving high-precision intelligent navigation. This paper proposes a data filtering and combined positioning method, and establishes an active screening model. The dynamic and static positioning drift points of the carrier are eliminated or replaced, reducing the complexity of the original Global Navigation Satellite System (GNSS) output data in the positioning system. Compared with the traditional Kalman filter combined positioning method, the proposed active filtering–Kalman filter algorithm can reduce the maximum distance deviation of the carrier along a straight line from 0.145 m to 0.055 m and along a curve from 0.184 m to 0.0640 m. This study focuses on agricultural robot positioning technology, which has an important influence on the development of smart agriculture.

## 1. Introduction

Agricultural robots use intelligent monitoring, automatic control, image recognition, environmental awareness, and other advanced technologies, as a whole, and, in horticulture, refinement of production, aquaculture intelligent production, plant protection precision operations, and other aspects, they play an important role in the realization of intelligent agriculture, precision agriculture, and unmanned agriculture as an important guarantee. At present, the most common navigation system for agricultural robots is a combination of the Global Satellite Navigation System (GNSS) and the Inertial Measurement Unit (IMU). However, complex and changeable environments affect the real-time positioning accuracy of agricultural robots, thus affecting their operating efficiency and accuracy [1,2,3,4,5,6,7,8,9,10]. Sun et al. [11] proposed a fast Kalman filter algorithm based on Upper–Downer (UD) covariance factorization and provided the hardware and software design and experimental results of the combined IMU/GPS system, which showed that the fusion localization accuracy was proven compared with single-sensor localization. Lu et al. [12] designed a combined navigation algorithm based on Rao-Blackwellized untraceable Kalman filtering (RB-UKF), which significantly improves the filtering accuracy of the combined navigation system after GPS signal interruption and restoration, and it is easy to implement in engineering. The fusion algorithm represented by Kalman filtering achieves higher positioning accuracy through data fusion between multiple sensors. In the combined positioning system, GNSS is used as an absolute positioning sensor, and its positioning data play a corrective role for the fused positioning point, during which the IMU continuously performs secondary integration with the previous GNSS positioning point as the starting point, thus estimating the position and compensating for the defects of missing positioning points during the GNSS signal return transmission. The effective fusion of the two can significantly improve positioning accuracy; however, most filtering algorithms can only passively receive sensor data during data fusion and do not directly screen and filter outliers [13,14]. This leads to the sensor measurement accuracy becoming the biggest factor affecting the fusion effect, given the absolute positioning role of GNSS in the filtering; when the GNSS positioning point drifts, the filtering effect is significantly reduced, and the positioning accuracy is significantly affected [15].

To address the problem of system accuracy degradation and poor navigation effect caused by GNSS positioning drift [16], Zhang et al. [16] proposed an extended Kalman filter algorithm that adaptively adjusts the system state covariance matrix to achieve real-time correction of the Kalman filter parameters. However, positioning errors accumulate over time and affect positioning accuracy. Compared to fusing different algorithms to process data, the method of eliminating outliers appears more in actual engineering applications. When the carrier is in a stationary state, the direct elimination method is more effective and can filter out most abnormal points. However, when the carrier is in motion, direct elimination will cause missing positioning points, resulting in discontinuous positioning problems, and positioning accuracy cannot be guaranteed [17,18]. To solve these problems, a combined positioning algorithm based on active screening–Kalman filtering is proposed. In this algorithm, the elimination and replacement of positioning drift points are accomplished; that is, the IMU is utilized to judge the carrier motion state, the static positioning drift points are eliminated, and then the static drift is suppressed within the allowable error range. Correspondingly, the same judgment conditions were used to mine the dynamic positioning drift points, and the Newton interpolation algorithm was used to replace the dynamic positioning drift points to reduce the dynamic drift within the permissible error range. Then, the position data after the positioning drift point elimination and replacement are input into the Kalman filter in real time, and the possible GNSS positioning oscillations and drifts are further corrected to obtain more accurate and smooth trajectory curves, which can provide more accurate positioning information for navigation operations. Based on the proposed algorithm, a C++&Python program deployed in Jetson Xavier NX|NVIDIA was developed by integrating the ROS system and was mounted on a two-wheeled differential servo chassis for comparative tests to measure the accuracy of the algorithm. The road experiment sections are divided into straight-line sections and curved sections, and the dynamic drift error of the straight line is reduced from 6.7619 cm to 2.7918 cm, and that of the curved line is reduced from 8.4296 cm to 4.6245 cm as the algorithm is applied.

## 2. Materials and Methods

**Figure 1:**
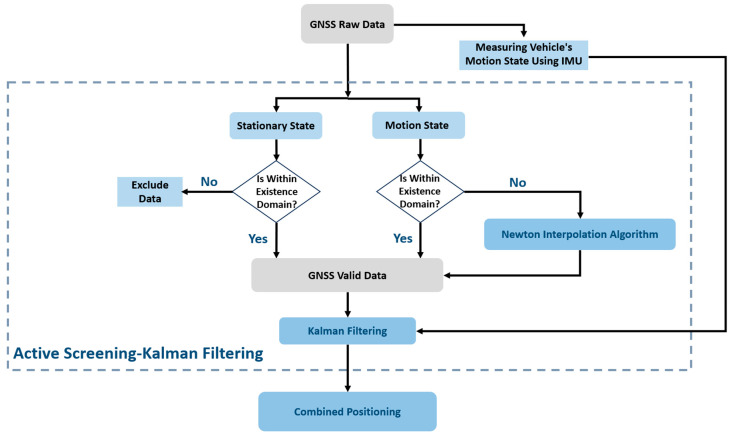
Active Screening–Kalman Filtering.

The algorithm flow chart of the active screening–Kalman filter proposed in this article is shown in Figure 1. First, the GNSS raw data were actively screened. During this period, the IMU measured the vehicle motion status in real time and used this as the basis for screening to determine whether the positioning was correct. The points were retained, eliminated, or replaced, and then the filtered valid GNSS data were used to perform Kalman filtering processing in conjunction with the IMU data to achieve fusion positioning.

IMUs typically include three-axis acceleration, three-axis angular velocity sensors, and magnetometers. Civilian-grade IMUs are affected by preparation processes and materials, and there are certain measurement errors. In order to ensure the accuracy of the data, this algorithm only uses the three-axis acceleration information measured by the IMU to avoid the sudden increase in errors caused by integration from affecting subsequent calculations. When using IMU to measure chassis acceleration, you first need to exclude the gravity component on the xyz axis. That is, after measuring the xyz three-axis acceleration, you need to subtract the acceleration component of gravity on the xyz three-axis. The gravity acceleration needs to be measured at the experimental site. Secondly, through the coordinate system conversion method, the IMU coordinate system is converted to the chassis geometric center position, and the measured chassis motion state can be obtained; that is, the three-axis acceleration value can be obtained.

Owing to the environmental impact or data transmission, drift cannot be avoided during the GNSS position information-receiving process, thus influencing real-time filtering processing. Therefore, drift points should be identified and rejected before the data are input into the Kalman filter. According to the motion states, positioning drift points can be categorized into static and dynamic points.

After using the IMU to obtain the three-axis acceleration *a_x_*, *a_y_*, and *a_z_* and angle information at time *t*, the area where the GNSS positioning point may appear at the next moment can be estimated; this is called the existence domain of the positioning point. Considering that the IMU may have a certain degree of error, after expanding the existence domain of positioning points appropriately, the points inside the domain are accepted into the subsequent mean value calculation process, and the points outside the domain are judged as drifting points and filtered out. As the carrier is in a stationary state, every moment will be included in the domain of the point for the calculation of the mean value, and once the carrier resumes movement, the mean value of the calculation results will become the actual position in the stationary state to participate in the subsequent operation. The coordinate position fed back by GNSS at time *t*_0_ is (*x_t_*, *y_t_*, *z_t_*), the line acceleration information fed back by the IMU is (*a*_*x*,*t*_, *a*_*y*,*t*_, *a*_*z*,*t*_), the angle information is (*θ*_*x*,*t*_, *θ*_*y*,*t*_, *θ*_*z*,*t*_), and the existence domain of the next GNSS coordinate point position is

(1) $$x_{t+1}=x_{t}+(t_{0}-t_{1})\int_{t_{0}}^{t_{1}}\bar{a_{x,\;t}}d\left(t\right)+\frac{1}{2}\bar{a_{x,t}}{\left(t_{0}-t_{1}\right)}^{2}$$

(2) $$\;y_{t+1}=y_{t}+(t_{0}-t_{1})\int_{t_{0}}^{t_{1}}\bar{a_{y,t}}d\left(t\right)+\frac{1}{2}\bar{a_{x,t}}{\left(t_{0}-t_{1}\right)}^{2}$$

(3) $$z_{t+1}=x_{t}+(t_{0}-t_{1})\int_{t_{0}}^{t_{1}}\bar{a_{z,t}}d\left(t\right)+\frac{1}{2}\bar{a_{z,t}}{\left(t_{0}-t_{1}\right)}^{2}\;$$

**Figure 2:**
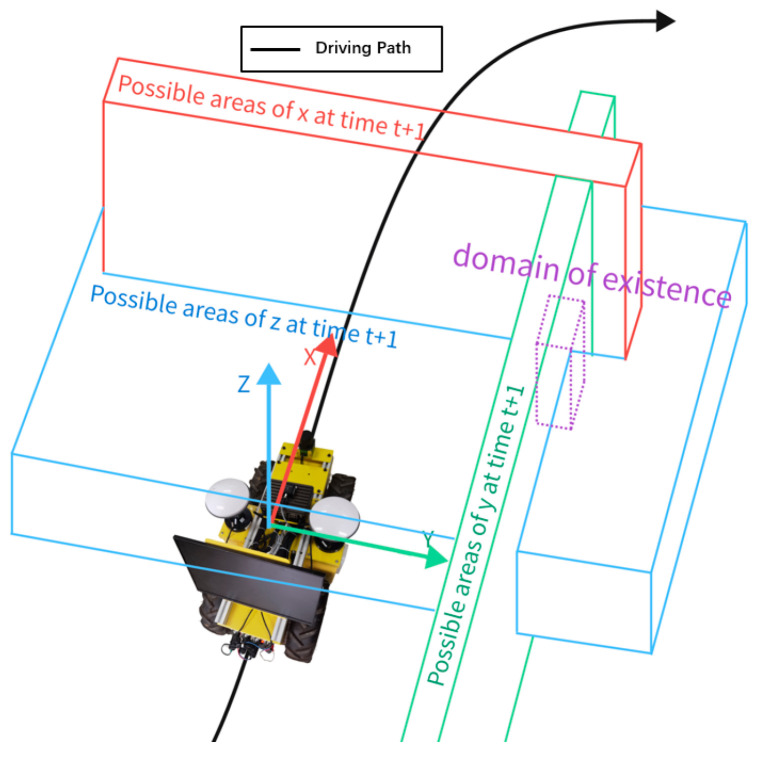
Existence domain.

As shown in Figure 2, the existence domain is a cubic region in the geometric sense, and a point set in the mathematical sense. Where *x*_*t*+1_, *y*_*t*+1_, *z*_*t*+1_ are the coordinates of the points at time of t+1, and considering the trace error in the three-axis acceleration measured by the IMU, the $\bar{a_{x,t}},\;\bar{a_{y,t}},\;\bar{a_{z,t}}$ can be extended to $a_{x,t}\pm \Delta a_{x},\;a_{y,t}\pm \Delta a_{y},a_{z,t}\pm \Delta a_{z}$; then, the existence domain is determined. As t+1, the coordinates of the GNSS position point at that moment fall within the existence domain, where the point is a valid positioning point; otherwise, it is an invalid drift point and rounded off. When the operating vehicle is in a stationary state, the three-axis acceleration measured by the IMU is extremely small, and by considering the paradigm of the IMU parameter matrix in the stationary state, the paradigm threshold can be obtained. An acceleration less than this threshold can be discarded for trace changes, and dynamic elimination of static positioning drift points can be achieved.

**Figure 3:**
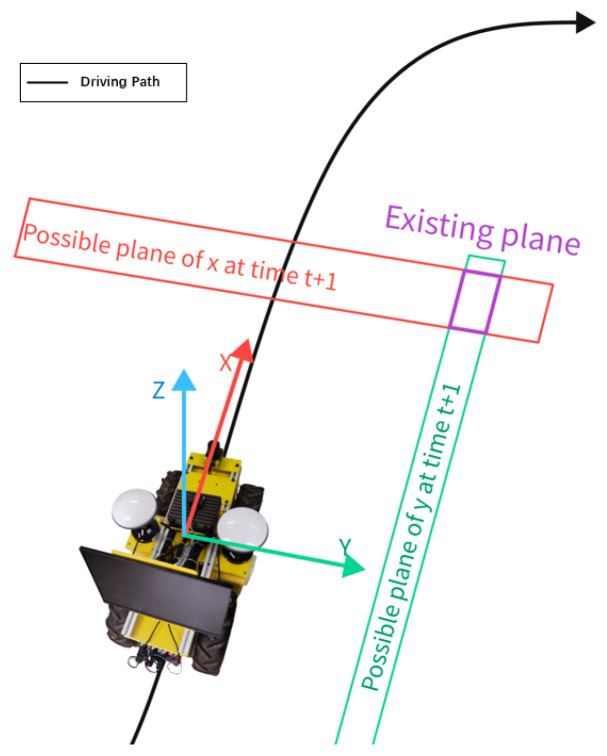
Rectangular existence surface.

In practice, GNSS is more effective in in-plane localization, and the localization accuracy is more affected by a larger change in elevation (altitude) [19]. For this reason, it is assumed that the operating vehicle is located at the same altitude for operation, or the change in elevation during operation is small; that is, the component of the GNSS data in the Z-direction in the carrier coordinate system is discarded. At this point, the cubic existence domain is reduced to a rectangular surface, as shown in Figure 3.

The screening process for positioning the drift points under the operating state of the vehicle was similar to that of the static positioning drift points. During the traveling process of the vehicle, the three-axis acceleration measured by the IMU is much larger than the static trace change, there is no superposition effect of the GNSS positioning points, and each positioning point is discrete and independent. According to the dynamic filtering method of static drift points, if only the points falling outside the presence domain are discarded, because the starting point for determining the presence domain is still the last positioning point that has not drifted, then the lack of new drifting positioning points will result in an inability to retain all subsequent positioning points. That is, if the anchor points drift at time t + 1, and do not drift at time t + 2, it can be expressed as

(4) $$x_{t+2}=x_{t+1}+\Delta x,\;\Delta x>0$$

(5) $$y_{t+2}=y_{t+1}+\Delta y,\;\Delta y>0$$

In addition, point (*x_t_*_+2_, *y_t_*_+2_) will not exist in the domain. Because the location of the vehicle constantly changes, using the positioning point at time *t* to judge the positioning point at time *t* + 2 cannot guarantee the accuracy of the positioning point at time *t* + 2, and the position information at time *t* + 1 is lost. Therefore, the Newtonian interpolation method was applied to determine the positional drift at time *t* + 1 replace the dynamic positioning drift points generated at time *t* + 1 to reduce the positioning error. And the longitude and latitude data of the positioning points before time *t* need to be obtained. Using time as a measurement, the longitude and latitude are divided into two dimensions to avoid the same longitude or latitude data appearing after the vehicle is stationary, causing fitting failure. 

Taking the longitude interpolation as an example, assuming that the current time is *t* and the node at this time is (*t*, *Lon_t_*), selecting the first *x* + 1 accurately localized points. In the Newton interpolation method, an interpolation polynomial of the same order as the number of interpolation nodes must be set to predict or interpolate the values within a specific range. Therefore, *P*(*t*) was set as a prediction polynomial. *T* is the time and *Lon* is the longitude; then, the polynomial to be found, *P_n_*(*T*), is

(6) $$P_{n}\left(T\right)=Lon_{t-x}+Lon_{t-x+1}T+\ldots +Lon_{t}T^{n}$$

where the n polynomial *P_n_*(*T*) of the number of times, then there are

(7) $$P_{0}\left(T\right)=Lon_{t-x}-x$$

*P*_0_(*t*) is a zero-order polynomial that describes a single point. Similarly, *P*_1_(*t*) is a first-order prediction polynomial describing a straight line. Let *Q*_1_(*T*) be the main function of the prediction polynomial. Then,

(8) $$P_{1}\left(T\right)=Lon_{t-x}+Q_{1}\left(T\right)$$

*P*_1_(*T*) simultaneously satisfy (*t* − *x*, *Lon*_*t*−*x*_) with (*t* − *x* + 1, *Lon*_*t*−*x*+1_) conditions when *T* = *t* − *x* when there is

(9) $$P_{1}\left(T\right)=Lon_{t-x}+Q_{1}\left(T\right)=Lon_{t-x}$$

At this point *Q*_1_*(T) =* 0, by the algebraic theorem

(10) $$Q_{1}\left(T\right)=\left[T-\left(t-x\right)\right]a_{1}$$

where *a*_1_ is a constant obtained in the subsequent derivation.

As T = t − x + 1, $a_{1}=\frac{Lon_{t-x+1}\;-\;Lon_{t-x}}{\;\left(t-x+1\right)\;-\;\left(t-x\right)}$ and Equation (10) can be expressed as

(11) $$\begin{array}{c} P_{x}\left(T\right)=Lon_{t-x}+\left[T-\left(t-x\right)\right]a_{1}+\left[T-\left(t-x\right)\right]\left[T-\left(t-x+1\right)\right]a_{2}\\ +\ldots +\left[T-\left(t-x\right)\right]\left[T-\left(t-x+1\right)\right]\ldots \left[T-\left(t-1\right)\right]a_{x} \end{array}$$

Different operating scenarios of vehicles will result in different planned paths, which makes the selection of the number of known points extremely important during interpolation calculations. Therefore, the order of the interpolation polynomial generally differs according to different paths. One of the advantages of Newton’s extrapolation method is that, when adding an interpolation node, one term can be added directly after the polynomial:

(12) $$P_{x+1}\left(T\right)=P_{x}\left(T\right)+\left[T-\left(t-x\right)\right]+\left[T-\left(t-x+1\right)\right]\ldots \left[T-\left(t\right)\right]a_{x+1}$$

This feature avoids the problem of recalculating the entire polynomial because of the change in the interpolation node in the Lagrangian interpolation method, which saves computational resources and is more efficient.

Interpolation with time as a node belongs to equidistant node interpolation, which is calculated after introducing the differential Δ. After introducing the concept of difference, the calculation can be obtained as

(13) $$a_{k}=\frac{\Delta^{k}Lon_{t-x}}{k!h^{k}}\left(k=t-x,\;\ldots ,\;t\right)$$

When the positioning point at time *t* + 1 drifts and falls outside the existence domain, an appropriate *x* value is selected based on the experimental results to calculate the interpolation polynomial, and *t* + 1 is introduced into the equation to obtain the longitude information at that time.

## 3. Algorithm Design for Combinatorial Localization Systems

The use of multi-sensor information fusion technology in a combined navigation system can extend the temporal and spatial coverage of the entire system, increase the information utilization of the system, improve the confidence and accuracy of the fused data, and enhance the fault tolerance and reliability of the system [7,8]. After active screening of the original data of the combined positioning system, the Kalman filter algorithm is used to process the data again to further correct the drift error of the positioning point. Before inputting the combined positioning system data into the Kalman filter for the positioning operation, it was necessary to convert the coordinate system of the GNSS data. Because the original GNSS output positioning information is based on the latitude, longitude, and elevation information of WGS-84 the World Geodetic System), in order to enhance its intuitive nature, the coordinate system is converted to XY plane coordinates to ensure a more intuitive and accurate positioning effect. This effect was intuitive and precise. The conversion of the coordinate system involves the conversion between two ellipsoids, and the parameters of the WGS-84 coordinate system are half-length axis *a* = 6,378,137 m. The parameters of the WGS-84 coordinate system are half-length axis, ellipsoid oblateness *f* = 1/298.257223563. The parameters of the WGS-84 coordinate system are half-length axis and ellipsoid oblateness. The ellipsoid oblateness can be expressed as

(14) $$f=\frac{a-b}{a}$$

The semi-short axis can be found b, by Equation (15):

(15) $$e=\sqrt{\frac{a^{2}-b^{2}}{a^{2}}}$$

The first eccentricity e of the ellipsoid can be determined, and the first auxiliary coefficient *W* is

(16) $$W=\sqrt{1-e^{2}\sin{\left(B\right)}^{2}}$$

Then, the radius of curvature N of the ellipse is

(17) $$N=\frac{a}{W}$$

The final XYZ coordinates are converted from WGS-84 coordinates, where BLH are the latitude, longitude, and height of the WGS-84 coordinate system (Breit, Länge, Höhe):

(18) $$X=\left(N+H\right)\times \cos B\times \cos L$$

(19) $$Y=\left(N+H\right)\times \cos B\times \sin L$$

(20) $$Z=\left[N\times \left(1-e^{2}\right)+H\right]\times \sin B$$

In a combined localization system, it is first assumed that the carrier moves only in the x-direction and that the state vectors $x_{k}={\left[d_{xk},v_{xk}\right]}^{T}$, that is, the displacement and velocity in the x-direction, are given by

(21) $$d_{xk}=d_{xk-1}+v_{xk-1}\cdot \Delta t+\frac{1}{2}\cdot a_{xk-1}\cdot \Delta t^{2}$$

(22) $$v_{xk}=v_{xk-1}+a_{xk-1}\cdot \Delta t$$

Integrating the above formulas:

(23) $$d_{xk}=\left[\begin{array}{cc} 1 & \Delta t\\ 0 & 1 \end{array}\right]{\left[d_{xk-1},v_{xk-1}\right]}^{T}+\left[\begin{array}{l} \frac{1}{2}\Delta t^{2}\\ \Delta t \end{array}\right]{\left[a_{k-1}\right]}^{T}+W_{k-1}$$

After expanding the single X-direction motion into XY-plane motion, the equation of state of the combined positioning system is as follows:

(24) $$\left[\begin{array}{l} d_{xk}\\ v_{x,k}\\ d_{yk}\\ v_{y,k} \end{array}\right]=\left[\begin{array}{cccc} 1 & \Delta t & 0 & 0\\ 0 & 1 & 0 & 0\\ 0 & 0 & 1 & \Delta t\\ 0 & 0 & 0 & 1 \end{array}\right]\left[\begin{array}{c} d_{xk-1}\\ v_{x,k-1}\\ d_{yk-1}\\ v_{y,k-1} \end{array}\right]+\left[\begin{array}{cc} \frac{1}{2}\Delta t^{2} & 0\\ \Delta t & 0\\ 0 & \frac{1}{2}\Delta t^{2}\\ 0 & \Delta t \end{array}\right]\left[\begin{array}{l} a_{x,k-1}\\ a_{y,k-1} \end{array}\right]+W_{k-1}$$

(25) $$\left[\begin{array}{l} d_{xk}\\ d_{yk} \end{array}\right]=\left[\begin{array}{cccc} 1 & 0 & 0 & 0\\ 0 & 0 & 1 & 0 \end{array}\right]\left[\begin{array}{c} d_{xk-1}\\ v_{x,k}\\ d_{yk-1}\\ v_{y,k} \end{array}\right]+V_{k}$$

The linear acceleration in all three directions *a_x_*, *a_y_*, and *a_z_* are given by direct measurements from the IMU.

The final combined positioning system model is obtained as follows:

(26) $$X_{k}=AX_{k-1}+Bn_{k-1}+W_{k-1}$$

(27) $$Z_{k}=HX_{k}+V_{k}$$

where *X_k_* is the state vector of the combined navigation system at moment *k*; *Z_k_* is the observation vector of the combined navigation system at moment *k*; *n_k_*_−1_ is the control input vector of the combined navigation system at moment *k* − 1; *A* is the state transfer matrix of the combined navigation system, representing the change in the system from the state at moment *k* − 1 to the state at moment *k*; *B* is the control input matrix of the combined navigation system, representing the transition matrix from the control input to the current state of the system; *H* is the observation matrix of the combined navigation system, representing the system state vector *X_k_* gain on the measured variables; *W_k_*_−1_ is process noise; and *V_k_* is observation noise.

Generally, the data output frequency of IMU is higher than that of GNSS equipment. In order to ensure the smooth progress of Kalman filtering, timestamps can be used to align GNSS and IMU data before starting the fusion. The IMU data can be down-sampled to the corresponding time interval based on the timestamp of the GNSS data to keep the two synchronized.

In the model, the process noise *W*_*k*−1_ and measurement noise *V_k_* were mutually independent, Gaussian white noise satisfied a normal distribution, and the mean value was set to zero. The covariance matrix of the process noise was *Q*, and the covariance matrix of the observation matrix was *R*. Through experiments, based on fixed points or fixed paths with known longitude and latitude, the output value of the Kalman filter was recorded when the chassis was running, and based on this, the values of the *Q* and *R* matrices in the Kalman filter were adjusted to obtain the best matrix. In this article, the *Q* matrix and *R* matrix are *Q* = 10^−3^ × [0.92, 0, 0, 0; 0, 1.05, 0, 0; 0, 0, 0.93, 0; 0, 0, 0, 1.05], *R* = 10^−2^ × [4.11, 0, 0, 0; 0, 3.85, 0, 0; 0, 0, 4.11, 0; 0, 0, 0, 3.84].

The Newtonian interpolation algorithm was modeled and validated using MATLAB 2020. After importing the n longitude information, with the number of selected interpolation points as a variable, from the interpolation starting point traversing to the endpoint of interpolation, the interpolation operation was carried out, and the interpolated value obtained from each calculation was compared with the accurate value to determine the interpolation error. After traversal, the overall error minimizes the overall error value, which is the most appropriate interpolating polynomial order in that trajectory state. The selected trajectory data have a total of 61 values. The data come from the open-source GPS data set named Geolife Trajectories1.3. Taking into account the limitations of positioning equipment, the positioning accuracy was maintained at the meter level. For this reason, the longitude information was only retained to the fourth decimal place. Table 1 gives some results of the interpolation simulation.

From the simulation data, it can be observed that there is no positive correlation between the order of the interpolated polynomial equations and interpolation accuracy; that is, as the number of interpolated nodes increases, the interpolation error does not decrease, which is evident in the 1st to 6th experiments. Therefore, the interpolation order was appropriately reduced to produce the results of the 7th to 9th experiments. Under the assumption that the data set localization information is accurate, the average error is 1.31 × 10^−4^, and the result is 0.799 × 10^−5^ after calculating and averaging all the remaining data. After replacing the data set by repeating several experiments, the average error stabilized in the range of [0.872 × 10^−5^, 0.451 × 10^−5^]. The selection of the optimal number of interpolation nodes is of great significance for further reducing the error. After analyzing the trajectory characterization, the trajectories in the data set can be roughly divided into two types of straight-line segments and curved segments for different morphologies. The optimal number of interpolation nodes corresponding to the two types of statuses is not exactly the same, and further judgment should be made according to the specific situation. Based on the above phenomenon, before active filtering–Kalman filtering is involved in the actual positioning and navigation operation, the road information should be collected, and the above experiments should be carried out to clarify the optimal number of interpolation nodes on different road sections, and then ensure that different road sections are well adapted in the actual operation process. The interpolation node-switching conditions under different road sections are provided by sensors such as IMUs, which can obtain heading information. The switching conditions of the interpolated nodes under different road sections are provided by sensors, such as IMUs, that can obtain heading information. The simulation experiment of the interpolation algorithm verifies the feasibility of the interpolation algorithm in trajectory prediction, thereby providing a theoretical basis for the replacement processing of dynamic positioning drift points in actual positioning operations.

The latitude information can be obtained by the same calculation, and, finally, a substitute value closer to the real value can be derived to participate in the determination of the localization point at the next moment, i.e., *t* + 2 moment, thus eliminating the influence of the direct elimination of the dynamic localization drift point on the whole localization process under the premise of guaranteeing the continual feasibility of the method of determining the existence domain.

## 4. Experiments and Discussions

**Figure 4:**
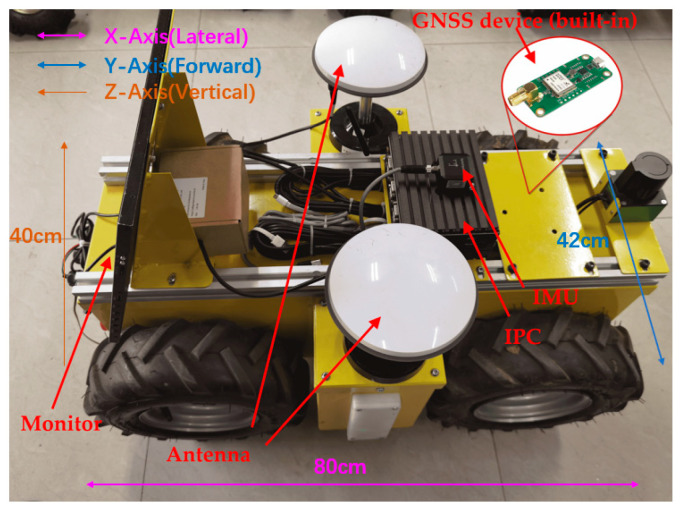
Test platform.

Figure 4 shows the test platform, which is designed as a four-wheel differential chassis and is equipped with an NVIDIA Jetson Xavier NX industrial computer, GNSS equipment (built-in), IMU equipment, and GNSS multi-frequency antennas. The four-wheel differential chassis uses a high-speed DC encoder motor and a worm gear reducer to provide walking power to improve field obstacle surmounting performance and ensure passability. At the same time, the reducer has a self-locking function, which can effectively reduce displacement errors caused by inertia. Considering that the chassis travels at a low speed during field operations, the maximum chassis speed is set to 0.5 m/s, and the maximum torque of the wheel set is 70 N·m. At the same time, the built-in motor encoder can provide accurate displacement and angle feedback information during driving. The main control platform is Jetson Xavier NX|NVIDIA, which has a computing power of up to 21 tops and can meet the needs of multi-sensor data fusion with frequencies less than 100 Hz. The GNSS equipment uses the HI600 board, the positioning frequency can be stably maintained at 5~10 Hz, and it has centimeter-level positioning accuracy after cooperating with RTK and GNSS multi-frequency antennas. The inertial measurement unit uses the Orientus module produced by the Australian Advanced Navigation Company, which can directly measure three-axis acceleration, angular acceleration information, and secondary data. The roll and pitch accuracy is 0.2° (static)/0.6° (dynamic), heading accuracy is 0.5° (static)/1.0° (dynamic), and the frequency is 1000 Hz, and the frequency is 1000 Hz. The chassis motor and its various sensors use CAN bus and ROS to communicate with the embedded platform to achieve multi-sensor fusion. Considering that the maximum driving speed of the chassis is low, in order to ensure enough sampling points and avoid fluctuations in the signal stability of the GNSS equipment caused by a long experiment time, it is necessary to select an appropriate driving speed for the chassis. After multiple tests, it was found that if the speed is too low, the experimental time will be extended, the possibility of being affected by the environment will increase, and the impact of equipment temperature rise on performance will be more significant. If the speed is too fast, there will be insufficient sampling points, and the experimental reliability will be compromised. Lower, for this purpose, the chassis is controlled to travel at a speed of 0.2 m/s for testing. In order to simulate the actual open-field environment as much as possible, the experiment was conducted at the outdoor experimental site of China Agricultural University. During the GNSS signal acquisition process, multipath effects and occlusion effects have a greater impact on positioning accuracy. The impact of multipath effects is mainly reflected in the reflection and refraction of GNSS signals due to clouds, lakes, building glass, etc., resulting in the actual propagation path of the signal. This is extended, and the impact of the blocking effect is reflected, in the signal strength being weakened by tall buildings, trees, etc., blocking the signal [20]. For this purpose, the experimental time was chosen from 8 a.m. to 11 a.m., during which the weather was sunny and there was little cloud cover. The experimental area has no obstructions above the horizon angle of 30° and no high-reflectivity materials around it, ensuring the accuracy of GNSS signals [21,22,23].

**Figure 5:**
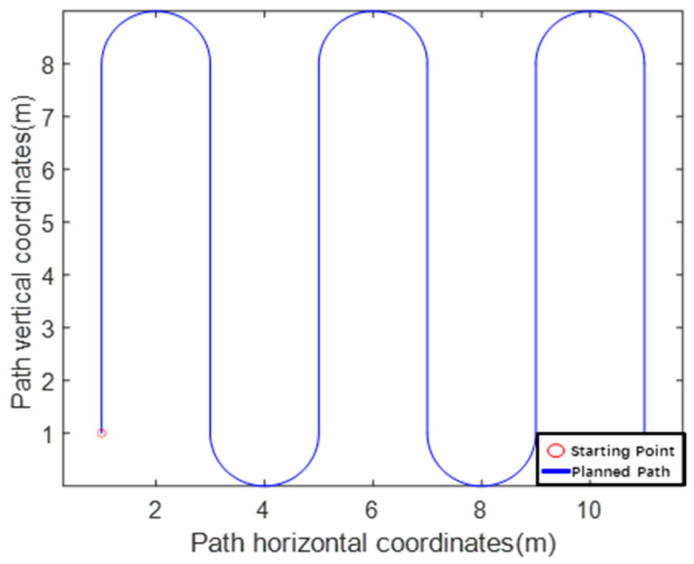
Artificially planned path.

**Figure 6:**
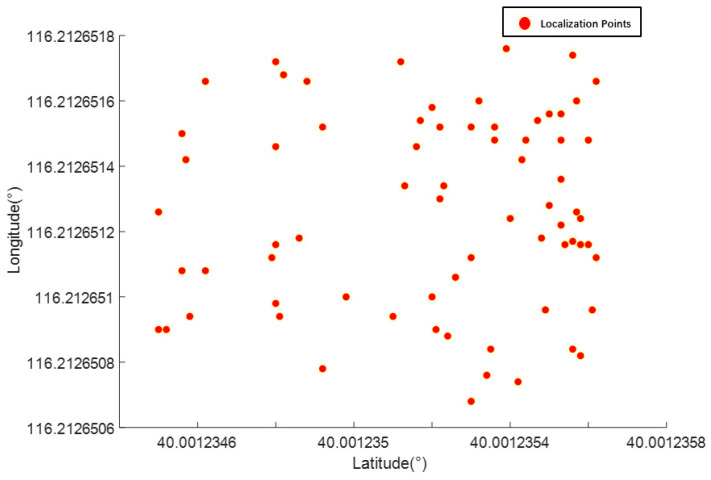
Distribution of sampling points.

Figure 5 shows the driving path of the experimental platform drawn using MATLAB, which starts at (1, 1) and ends at (9, 1). The path feature points were extracted, as shown in Table 2. Prior to the static test, the current position of the experimental platform was localized. The experimental platform was placed in the environment for 155 min and differential RTK technology was used to perform positioning point sampling at intervals of 4 s. Seventy representative sampling points were selected for the calculations. Figure 6 shows the distribution of the sampling points, in which the minimum value of latitude was 40.001235, maximum value was 40.001236, minimum value of longitude was 116.212651, and maximum value was 116.212652. The average value of latitude was 40.001235 and that of longitude was 116.212651. The average values of latitude and longitude were used as the starting points of the experimental platform to participate in the experiment.

To consider the effect of the algorithm more intuitively, the error expressed in latitude and longitude was converted to a distance in meters. The radius of the earth is known as

(28) R=6371.004 km

(29) $$L_{E}=\frac{\pi R}{180}=111.195\;\mathrm{km}$$

(30) $$L_{N}=\frac{\pi \mathrm{Rcos}\theta }{180}=85.176\;\mathrm{km}$$

where *θ* is the latitude of the localization point.

*L_E_* is the actual difference in distance between one degree of latitude on the same longitude line.

*L_N_* is the actual difference in distance between one degree of longitude on the same latitude line.

**Figure 7:**
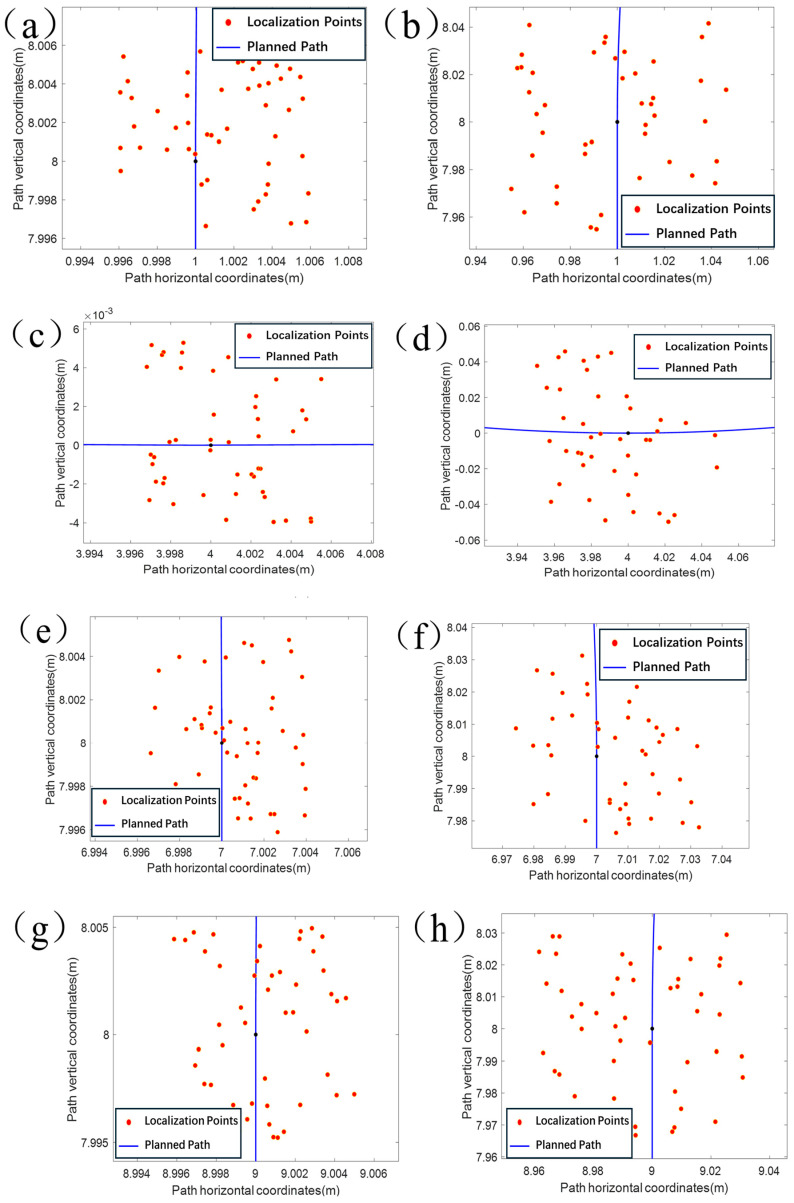
Static positioning drift test results.

After determining the longitude and latitude coordinates of the starting point of the experimental platform, correspond them to the starting point of the planned path (1, 1), and convert the path feature points to WG-S84 coordinates. While the experimental platform traveled along the planned path, the static positioning point distribution after braking was collected multiple times in the straight and curved paths to analyze the suppression of drift by the active filtering–Kalman filtering algorithm in a static state. After removing the starting point, the 2nd, 6th, 10th, and 14th feature points were selected, and the chassis remained at each point for 30 min to take samples. Figure 7a–d show the static test results. The planning paths are the blue straight line and curved segments, and the red points are the localization points. It can be found that the static maximum drift in the X-direction is 0.0049 m, and the minimum drift is 0.00020 m; the static maximum drift in the Y-direction is 0.0048 m, and the minimum drift is 0.00040 m after active filtering–Kalman filtering algorithm processing. The average drift in the X-direction was 0.00030 m and the average drift in the Y-direction was −0.0004 m. The maximum distance deviation in a single direction was only 0.00040 m, the maximum filtering radius was 0.0048 m, and the standard deviation of the distance deviation was 0.0028 m. Figure 7e–h show the results of the data output of the Kalman filtering algorithm without active filtering. When the raw data of the combined navigation device were not actively filtered, the static maximum drift in the X-direction was 0.05000 m, minimum drift was 0.00020 m, static maximum drift in the Y-direction was 0.04900 m, minimum drift was 0.00070 m, average drift in the X-direction was 0.0021 m, average drift in the Y-direction was 0.00130 m, average drift in the Y-direction was 0.00210 m, average drift in the Y-direction was 0.00130 m, and average drift in the Y-direction was 0.00130 m. The maximum distance deviation in a single direction was −0.00130 m, the maximum filter radius was 0.05000, and the standard deviation of the distance deviation was 0.03800 m. It can be observed that the active filtering–Kalman filtering algorithm is better than Kalman filtering alone in static localization, and it can provide more accurate position information for static localization by comparing the two data.

**Figure 8:**
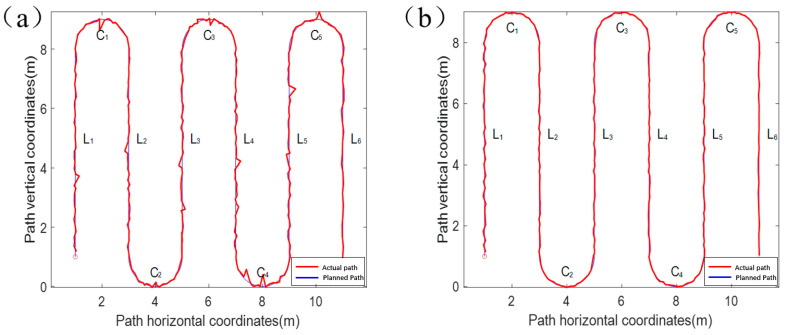
Dynamic positioning drift test results: (**a**) the test without the Kalman filter algorithm; (**b**) the test with the active screening–Kalman filter algorithm.

**Figure 9:**
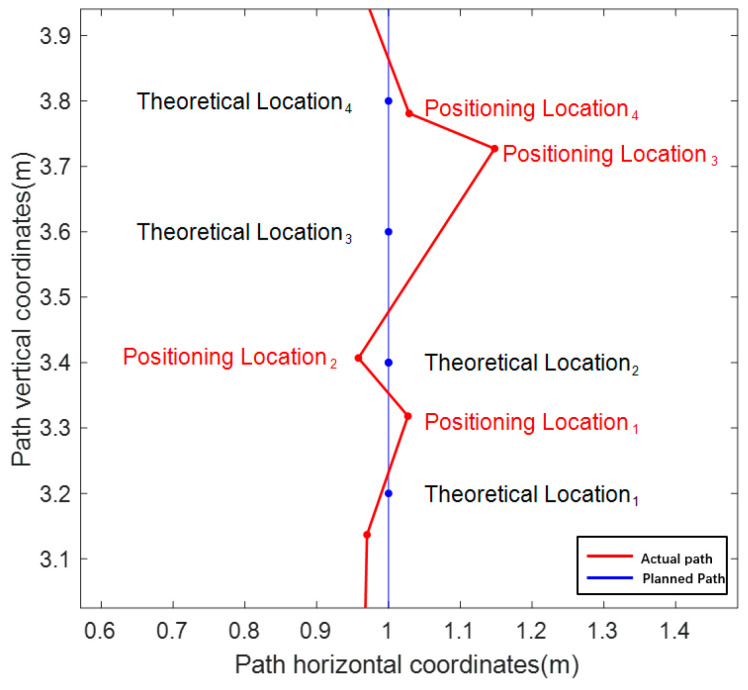
Theoretical and actual positions.

The localization data in the motion state are shown in Figure 8, and Figure 8a shows a comparison between the Kalman-filtered localization point data (red curve) without active screening and the planned path. When the original data of integrated navigation are not automatically filtered, outliers will appear in positioning owing to equipment frequency jitter, atmospheric refractive index changes, etc., and these outliers will have a greater impact on the positioning effect. The errors in the linear and curved segments of the planned path were considered separately to facilitate the measurement of the results. Figure 8a shows the straight-line segment, which is defined as *L*_1_–*L*_6_, and the curved segment is defined as *C*_1_–*C*_5_. Under the premise that the frequency of the combined positioning equipment, speed of the experimental platform, and length of each section of the path are all determined, each positioning point has a corresponding “theoretical position,” that is, the position that should appear if the positioning does not drift, as shown in Figure 9. During the dynamic positioning process, it is obvious that the drift in a certain direction alone cannot accurately measure the positioning error because the drift errors in both XY directions are not of the same order of magnitude. For these reasons, the distance between the theoretical position and the fixed position is taken as the distance deviation, and the distance deviation and standard deviation of the distance deviation for each segment are listed in Table 3.

In the absence of active screening, the average distance deviation of the straight-line segment route was 0.0676 m, the maximum distance deviation was 0.1452 m, and the standard deviation of the maximum distance was 0.2423 m. The calculation results of the curved segment are listed in Table 3, where the average drift error is 0.0840 m, the maximum distance deviation is 0.1836 m, and the standard deviation of the maximum distance deviation is 0.2600 m. Comparing the straight-line segment with the curved segment, it can be seen that the curved segment is more prone to anomalies, which in turn leads to an increase in the average distance deviation of the data, as well as the standard deviation. It can be observed that different curvatures lead to different effects on the results of locating anomalies. Figure 8b shows the results of processing the raw data of combined navigation using active screening–Kalman filtering (red curve) compared with the planned path, and no obvious anomalies can be found in the data processed by active filtering–Kalman filtering; this is due to the dynamic positioning drift point replacement step, which discards points that appear outside the existence domain. Similarly, after analyzing the straight-line segment positioning points and curved segment positioning points processed by active screening and Kalman filtering, it was found that the average distance deviation of the straight-line segment was 0.028 m, the maximum distance deviation was 0.055 m, and the maximum distance deviation standard deviation was 0.033 m. The calculation results of the curved segment show that the average drift error is 0.0460 m, the maximum distance deviation is 0.0640 m, and the maximum distance deviation standard deviation is 0.0620 m. 

Through the analysis of the two positioning results, the active filtering–Kalman filtering is better than the Kalman filtering without active filtering in optimizing the positioning of both linear and curved segments. Under the effect of active filtering–Kalman filtering, the average distance deviation of the straight-line segment was reduced by 0.04 m, with an error reduction of 59%, and the average distance deviation of the curved segment was reduced by 0.038 m, with an error reduction of 45%. However, when the driving condition is complicated, and several consecutive curves with large headings change, the positioning accuracy may be significantly decreased; however, the overall positioning accuracy is significantly improved. The main reason for the difference in positioning accuracy between the straight line and curved section is that the IMU always lags behind the actual operation of the vehicle. After the vehicle has been displaced for a certain period of time, the IMU can judge that the vehicle is turning through a change in the heading angle, and then adjust the nodes selected by interpolating polynomials to fit the curved road paths. The lag effect of IMU’s perception of the vehicle’s motion state also occurs at the junction of turning and going straight, after the end of the turn, where the number of interpolated nodes cannot be corrected in real time to the number of nodes suitable for the straight path, resulting in a large change in the path or vehicle heading within a short period of time, resulting in a decrease in the localization accuracy, and the sensitivity of the interpolation algorithm to the interpolated nodes is also a cause of this problem. Therefore, the positioning error can be reduced by reducing the driving speed of the vehicle when its driving state is about to change, increasing the sensing time of the IMU, and reducing the number of nodes to correct the time lag with vehicle operation. Similarly, for the complex continuous heading change problem, the interpolation algorithm can be corrected according to the carrier’s motion equation. At the same time, the integral characteristics of the IMU can be used to project the information about the displacement and corner change of the carrier in a very short period to provide more accurate judgment information for the substitution of the dynamic localization drift point.

## 5. Conclusions

This paper proposes an active screening–Kalman filter algorithm that utilizes the IMU to sense the robot’s motion state and combines it with the kinematic algorithm to determine the localization points dynamically. An interpolation algorithm is applied to replace the rejected localization points to ensure the sustainability of the trajectory. The following conclusions can be drawn:

1. A combined positioning algorithm based on active filtering and a Kalman filter is proposed, which completes the elimination and replacement of GNSS positioning drift points and the correction of drift, reduces the impact of GNSS drift on the filtering effect, and improves positioning accuracy.
2. An integrated control system was built using Python and the Jetson Xavier NX|NVIDIA platform. GNSS dynamic and static positioning drift points are eliminated or replaced to implement data filtering through active screening models, and the filtered data are fused using Kalman filtering.
3. Experiments were carried out to verify the algorithm and the experimental results indicate that the static drift error of the combined navigation in a single direction is reduced from 1.34 to 0.43 cm, the dynamic drift error of the straight-line segment is reduced from 6.76 cm to 2.79 cm, and the curved segment dynamic drift error is reduced from 8.43 cm to 4.62 cm when the active screening–Kalman filter algorithm is applied, which proves that the active screening link can provide dynamic and reliable results.

## Figures and Tables

**Table 1:** Table of results of interpolated simulation section.

Interpolation Start Point	Number of Nodes	Interpolation Results	Inaccuracies	Interpolation Start Point	Number of Nodes	Interpolation Results	Inaccuracies
1	2	39.9899	0.00090	5	2	39.9891	0.00003
	3	39.9926	−0.00177		3	39.9884	0.00025
	4	39.9883	0.00252		4	39.9886	−0.00100
	5	39.9938	−0.00390		5	39.9845	0.00237
	6	39.9825	0.00670		6	39.9905	−0.00453
	7	40.0001	−0.01145		7	39.9774	0.00780
	8	39.9700	0.01768		8	39.9970	−0.01315
2	2	39.9917	−0.00087	6	2	39.9884	0.00028
	3	39.9900	0.00075		3	39.9884	−0.00075
	4	39.9913	−0.00138		4	39.9855	0.00137
	5	39.9864	0.00280		5	39.9881	−0.00217
	6	39.9934	−0.00475		6	39.9819	0.00327
	7	39.9814	0.00623		7	39.9893	−0.00535
	8	39.9917	−0.00488		8	39.9725	0.01068
3	2	39.9909	−0.00012	7	2	39.9881	−0.00047
	3	39.9906	−0.00063		3	39.9862	0.00062
	4	39.9877	0.00142		4	39.9867	−0.00080
	5	39.9906	−0.00195		5	39.9841	0.00110
	6	39.9862	0.00148	8	2	39.9867	0.00015
	7	39.9855	0.00135		3	39.9861	−0.00018
	8	39.9947	−0.00872		4	39.9849	0.00030
4	2	39.9907	−0.00075		5	39.9849	−0.00098
	3	39.9884	0.00078	9	2	39.9860	−0.00003
	4	39.9892	−0.00053		3	39.9850	0.00012
	5	39.9881	−0.00047		4	39.9846	−0.00068
	6	39.9840	0.00283		5	39.9809	0.00227
	7	39.9933	−0.00737				
	8	39.9700	0.01517				

**Table 2:** Path feature point information.

Serial Number	Coordinate X Value (m)	Coordinate Y Value (m)	Serial Number	Coordinate X Value (m)	Coordinate Y Value (m)
1	1	1	10	7	8
2	1	8	11	7	1
3	2	9	12	8	0
4	3	8	13	9	1
5	3	1	14	9	8
6	4	0	15	10	9
7	5	1	16	11	8
8	5	8	17	11	1
9	6	9			

**Table 3:** Test distance deviation results.

Straight-Line Segment	L1	L2	Curved Segment	C1	C2
Maximum distance deviation	0.1353	0.1257	Maximum distance deviation	0.1836	0.1235
Average distance deviation	0.0785	0.0753	Average distance deviation	0.0823	0.0927
Standard deviation of distance deviation	0.2113	0.2033	Standard deviation of distance deviation	0.2423	0.1633
straight-line segment	L3	L4	curved segment	C3	C4
Maximum distance deviation	0.1314	0.1296	Maximum distance deviation	0.1365	0.1933
Average distance deviation	0.0712	0.0691	Average distance deviation	0.0632	0.1022
Standard deviation of distance deviation	0.2423	0.2015	Standard deviation of distance deviation	0.1723	0.2613
straight-line segment	L5	L6	curved segment	C5	
Maximum distance deviation	0.1452	0.0822	Maximum distance deviation	0.1652	
Average distance deviation	0.0603	0.0513	Average distance deviation	0.081	
Standard deviation of distance deviation	0.1926	0.1326	Standard deviation of distance deviation	0.1124	

## Data Availability

The authors confirm that the data supporting the findings of this study are available within the article.
